# Evaluation of a cancer patient navigation program (“Onkolotse”) in terms of hospitalization rates, resource use and healthcare costs: rationale and design of a randomized, controlled study

**DOI:** 10.1186/s12913-018-3226-3

**Published:** 2018-06-05

**Authors:** Ralf Porzig, Sina Neugebauer, Thomas Heckmann, Daniela Adolf, Peter Kaskel, Ursula G. Froster

**Affiliations:** 1 ”Onkolotse“ Cancer Patient Navigation Project, Sächsische Krebsgesellschaft e.V, Schlobigplatz 23, 08056 Zwickau, Germany; 2 MSD SHARP & DOHME GMBH, Lindenplatz 1, 85540 Haar, Germany; 3 StatConsult Gesellschaft für klinische und Versorgungsforschung mbH, Halberstädter Strasse 40a, 39112 Magdeburg, Germany

**Keywords:** Oncology, Psychosocial care, Patient-relevant results, Disease costs, Treatment outcomes, Healthcare research, Healthcare competence, Disease management

## Abstract

**Background:**

Concepts for the nursing and care of cancer patients through a “navigation service” have attracted much interest. However, there is still room for improvement in terms of their funding and coverage. The Saxon Cancer Society designed a prospective, randomized, multicenter, longitudinal study with a view to determining the positive effects of a cancer patient navigator program. The objective of this ongoing study is to evaluate the impact of the cancer patient navigation program on cancer patients and cost bearers in Germany.

**Methods:**

The study population in this evaluation comprises cancer patients with gastric carcinoma, pancreatic carcinoma, colorectal cancer, melanoma or gynecological cancer who have been hospitalized at least once at one of the study centers as well as their relatives, outpatient and inpatient physicians, and cancer nurses. It is planned to randomize 340 cancer patients (stomach, colonic/rectal cancer, gynecological cancer, melanoma) at five centers to an intervention group (care by patient navigators based on standardized operating procedures) or a control group in a one-to-one ratio. The primary target parameter is the number of hospitalizations within the 12-month intervention period. The participants are asked to complete various questionnaires on patient-related outcomes at baseline and at 3 and 12 months (SF 36, HADS, PAM 13, and others). Data on drug therapy, utilization of health services, and medical expenses will also be analyzed.

**Discussion:**

For the first time, the study will provide data on the effectiveness of a patient support program in cancer care in Germany from a randomized trial with a high level of evidence.

**Trial registration:**

The study has been registered under DRKS00013199 in the German Clinical Trials Register.

## Background

The diagnosis of cancer has far-reaching and life-changing consequences for patients [1]. Despite the prevalence and paramount importance of cancer, our healthcare system is often inadequately prepared to address the needs of patients and their families in terms of psychosocial care, information provision, and coordination of necessary measures.

A patient navigation service for cancer patients was set up for the first time in the United States about fifteen years ago. Its aim was to provide timely access to high-quality care in a manner tailored to each patient [2–4]. The US National Cancer Institute extended the concept to include additional goals for selected cancer types, notably the improvement of patient satisfaction and cost effectiveness [5].

The qualification profile (medical training and/or nonmedical staff), the optimum duration of support [6], and the cancer patient navigation service activities required to meet the tasks at hand are still poorly standardized [7]. Only three randomized studies, all in the US and Canada, have so far been published on the effectiveness of the programs, and the results have been mixed [8–10].

In Germany, too, the data situation is very sketchy. However, an evaluation study on patient support of a health insurance fund with a quasi-experimental design which took into account various disease groups including cancer showed positive effects with regard to customer satisfaction, effectiveness/quality, efficiency, and system acceptance [11].

Against this backdrop, the Saxon Cancer Society (SKG) launched the “Onkolotse” cancer patient navigation program in 2009. It aims to provide healthcare professionals with effective support in their cancer-related work and to improve the care of cancer patients [12].

### Program description

“Onkolotse” cancer patient navigation program specialists are trained, SKG-certified nurses/carers, professionals, counselors, psychologists or social workers working in the field of oncology [13]. They are extensively trained in special self-paid courses that are divided into seven modules and comprise over 130 h of tuition. Training topics include issues relevant to cancer patients, in particular structures, processes, and contact persons in the management of the disease. The “Onkolotse” cancer patient navigator program stands for trustworthy information, support, and advice rooted in understanding and care, as well as psychological issues relating to cancer treatment [13]. Patients and their family members are assigned a skilled, permanent contact person to whom they can address their questions during the entire duration of cross-sectoral treatment and who also has the necessary time to devote to them. Care does not end with the acute phase of the disease but continues throughout rehabilitation and aftercare. Topics of the consultation can be, for example: which hospitals provide interventions or rehabilitation measures, what support services are available from authorities or health insurance funds (e.g. severe disability status, aids, rehabilitation), and who is able to provide outpatient aftercare. The knowledge gain, the support in developing or maintaining a positive attitude, and the teaching of problem-solving skills increase the individual’s health literacy and enable active, independent participation of the patient in the treatment process. Overall, continued support can help patients and relatives to cope better with a cancerous disease and to feel more at ease within the healthcare system.

### Objectives and hypotheses

The primary objective of this study is to evaluate the impact of the cancer patient navigation program on cancer patients and cost bearers in Germany. It is intended to provide decision-makers with helpful information that facilitates the transfer of the program to routine care.

#### Primary target parameter

The primary aim is to investigate changes in hospitalization rates of cancer patients.

The underlying hypothesis is that participation in the cancer patient navigation program is able to reduce the number of patients who need to be re-hospitalized within 12 months. The hospitalization rate (defined as at least one night spent in hospital within a 12-month follow-up period) is estimated to be 25% in the control group and 12.5% in the intervention group.

#### Secondary target parameter

The secondary target parameter is whether the control group and the intervention group differ in their HADS-D scores as a surrogate of psychological stress.

The underlying hypothesis is that the patients’ psychological burden is reduced by participation in the patient navigation project. Patients in the intervention group will have a lower level of anxiety and stress on the HADS-D scale compared to baseline (t1) and will have a lower level at three and 12 months compared to the control group.

#### Other secondary and explorative target parameters

Other target parameters will be compared between the intervention group and the control group. They include:

- The patients’ quality of life
- The relatives’ quality of life
- The psychological burden on relatives
- Health status / general sense of health
- Social support / resources of patients
- The patients’ health literacy
- Joint decision-making between the patient and physician
- Days absent from work
- The waiting time for required treatments
- Compliance with treatments
- Double tests
- Satisfaction with the treatments
- Workflows / operational processes
- Use of services
- Healthcare costs

In addition, explorative analyses are planned, including analyses of the type of cancer and other patient-relevant variables.

## Methods/design

In line with the primary study objective, the design was chosen so that evidence of the effectiveness of the intervention can be generated at the highest possible level of evidence. The project was therefore designed as a prospective randomized, longitudinal, multicenter study. To our knowledge, no other RCT has been conducted in Germany on this topic. Other groups used a quasi-experimental design in which the control group was generated from insurance data [11]. A multicenter approach was chosen to obtain larger sample sizes and to ensure that the study optimally represents cancer centers. In addition, various types of cancer will be considered to assess the effectiveness of the program across multiple indications.

The program was implemented at five centers in Saxony, Germany. The evaluation also takes into account the network partners (office-based physicians) of the specialized hospitals, insofar as they contribute patients to the program.

Eligible patients are identified during their hospitalization in the participating centers and are informed about the study in a personal interview and with the help of written documents. If the patients give their written consent, they are randomized to the intervention group or the control group on a one-to-one basis. They are randomized with regard to the center, the type of cancer, gender, and age group. The following cancers are documented: pancreatic, gastric, colonic/rectal carcinoma, gynecological cancer and melanoma. While patients in the control group receive standard care, those in the intervention group are also in regular contact with their patient navigator.

The observation period in the intervention (cancer patient navigation program participation) and control (no cancer patient navigation program participation) groups is 12 months per patient. In this time period about 20 talks with the cancer patient navigation program specialists shall be held.

**Fig. 1:**
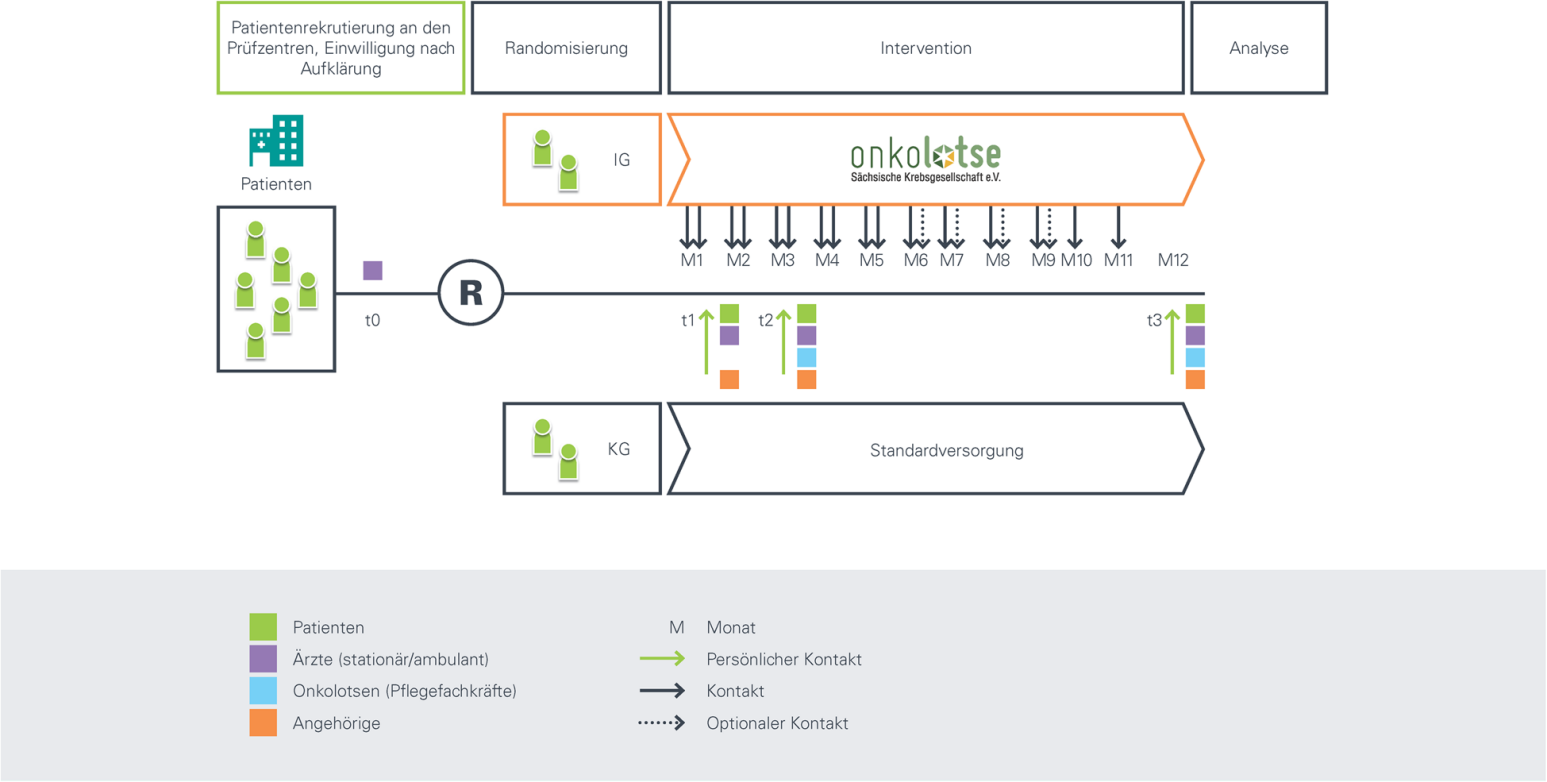
Study procedure. The figure shows the study flow from informed consent to randomization, intervention and analysis. Green arrows show personal contacts, black arrows any contact, dashed arrows optional contacts between study participants. M = month, R = randomization, t = time point#. Box colors: Green = patients, purple = physicians, blue = cancer patient navigators, orange = family members

Figure 1 summarizes the study design and the times of data collection. A distinction is made between the recruitment phase, the inclusion phase, and the three survey dates during which data are collected. Exams are scheduled during hospitalization (t1), after three months (t2), and after twelve months (t3). The data are provided by the physicians at the hospitals, the participating office-based physicians, the relatives of the patients, and the cancer patient navigation program specialists (only in the intervention group). Secondary data used in the study include internal hospital statistics provided by the participating centers (medical controlling).

### Study population

The study population comprises cancer patients who have been hospitalized at least once at one of the centers participating in the study as well as their relatives, outpatient and inpatient physicians, and cancer patient navigation program specialists. The inclusion and exclusion criteria for participation in the study must be observed.

Relatives of patients may be included regardless of their degree of relatedness, provided that they are of legal age and have declared their written consent to participate in the study. However, patients should nominate a close family member. Inclusion criteria for hospital physicians are that they must be employed at the hospital and have completed a good clinical practice course. Office-based physicians must be network partners of one of the participating hospitals. Participating cancer patient navigation program specialists must have successfully completed the SKG “Onkolotse” training course, have worked for at least two years in nursing at a cancer unit, and have cared for at least ten patients in their capacity as nurse navigation specialist prior to the study.

Neither the participating physicians nor the cancer patient navigation program specialists may have familiar relationships with the participating patients.

At least 340 cancer patients will be included in the study. The aim is to include not more than one family member per patient, although the exact number is not specified. Nor are the numbers of hospital-based and office-based physicians defined in advance.

### Inclusion criteria for patients

The patients must meet all of the following criteria to be included in the study:

- The patient is hospitalized at one of the five study centers.
- The patient has a diagnosis of gastric cancer, colonic/rectal cancer, pancreatic cancer, gynecological cancer, or melanoma.
- The patient has a cancer that falls into one of the following UICC TNM classifications:
- 0 (t in situ, N0, M0)
- Ia (T1, N0, M0)
- Ib (T2, N0, M0)
- IIa (T3, N0, M0)
- IIb (T4, N0, M0)
- IIIa (every T, N1, M0)
- IIIb (every T, N2, M0)
- IV (every T, every N, M1)

or has a recurrent tumor or metastases (in the case of melanoma, also metastases that do not affect the skin).

- The patient is at least 18 years old.
- The patient has signed the written informed consent form.
- The patient participates in the study voluntarily.

### Exclusion criteria

- The patient is permanently in an inpatient facility (for example palliative care or a hospice) for a terminal condition.
- The patient has a severe comorbidity whose treatment takes priority.
- The patient is in a disease management program.
- The patient is pregnant or breastfeeding.

### Measuring instruments and variables

The variables recorded in the study and the instruments used for that purpose are explained below:

#### Number of hospitalizations (primary endpoint)

This information is obtained by surveying the patients and also provided by the participating centers (medical controlling). Hospitalization is defined as a hospital stay which includes at least one overnight stay any time after the start of the study.

#### Psychological stress (secondary endpoint)

The Hospital Anxiety and Depression Scale (HADS) is validated for somatic diseases, for example, cancers of all indications, stages, and treatments [14, 15]. The instrument measures the patient’s psychological stress (anxiety and depression) during the previous week. It consists of 14 questions, each with four response categories. 0–3 points are awarded per response, and the sum score is calculated for each patient. A score of less than seven points means an unsuspicious result, a score of 8–10 points a suspicious result, and a score of over ten points a highly suspicious result. The scale is available in a validated German version [16]. The test has an internal consistency (Cronbach’s alpha) of 0.80, a test-retest reliability of *r* > 0.80 and good convergent and discriminant validity.

#### Quality of life of patients

The 36 items in Short Form 36 (SF-36) provide a validated cross-disease (generic) measurement tool for assessing the health-related quality of life of patients, which is also used in routine clinical practice [17]. The questionnaire captures eight dimensions of subjective health: Physical functioning, Physical role function, Physical pain, General health perception, Vitality, Social functioning, Emotional role function and Mental well-being; they can be assigned to the basic dimensions of physical and mental health. With a single item, the current state of health is also inquired compared to last year [18].

#### Question concerning subjective health perception

This validated instrument measures patients’ general subjective health status with just one question [19, 20], with five response options being given: 1 = “very good”, 2 = “good”, 3 = “moderate”, 4 = “poor”, 5 = “very poor”. A higher value indicates poorer health. The instrument is recommended by the World Health Organization and is also used in large-scale studies such as the European Health Interview Survey (EHIS).

#### Quality of life of relatives

This is being represented by subjective health perception questionnaire and HADS.

#### Social support/resources

The MOS Social Support Survey (SSS) is a short questionnaire for patients with chronic diseases [21]. Patients are asked to answer 19 questions describing four areas: emotional/informal support, measurable support, subjective support, and positive social interaction. There are five response options to each question. A cumulative score (social support index) is then calculated. For all areas, Cronbach’s alpha is > 0.91. The questionnaire has a high degree of convergent and discriminant validity.

#### Health literacy of patients

The HLS-EU 16 is the short version of the 47-question European Health Literacy Survey Tool [22, 23]. It covers the areas of knowledge, motivation, competency in gaining access to health services, and the ability to understand and appreciate information pertaining to healthcare, the prevention diseases, and the promotion of health. There are five response options for each of the 16 questions. A cumulative score is obtained for each patient which is classified into one of three categories: insufficient health literacy (range 0–8), problematic health literacy (9–12), and sufficient health literacy (13–16). Cronbach’s alpha for the score is 0.88.

#### Patient activity

The Patient Activation Measure (physicians’ version of PAM-13) allows a patient’s self-evaluation to be assessed [24]. Each of 13 questions has five response options (from 1 = “I strongly disagree” to 4 = “I strongly agree” and 5 = not applicable). Responses are converted to a score ranging from 0 to 100, with low scores indicating a low degree of patient activity. Cronbach’s alpha for the score is 0.80.

#### Joint decision-making

The questionnaire on participatory decision-making for chronic diseases (PEF-FB) consists of nine questions, each of which is answered on a six-point scale [25]. For each physician, the mean is calculated and converted into a standardized participation value ranging from 0 to 100 (with higher values indicating better participation). Cronbach’s alpha for the questionnaire is 0.88.

#### Days of work missed

The number of days from the start of the study on which the patient was unable to work − as confirmed by a medical certificate − is recorded. The details of the patients are documented on a specially created questionnaire.

#### Waiting time for required treatment

The waiting time is measured in days and is based on information provided by the patients themselves.

#### Compliance with treatment

Information on the patient’s compliance with the treatment is recorded by the physician on a standardized questionnaire. In cases where treatment is stopped, the causes are documented.

#### Doublication of tests

The medical controlling and network partners provide information on this subject on standardized questionnaires. Imaging and other diagnostic procedures are included.

#### Satisfaction with the intervention

The satisfaction of patients, physicians and relatives with the intervention program is recorded in questionnaires on a 5-point Likert scale (from “strongly agree” to “strongly disagree”).

#### Workflow/operative processes

Information on time spent with the patient, perceived time saving, improvements in physician-patient communication, and subjective evaluation of the effectiveness of the intervention is solicited in questionnaires and rated on a five-point Likert scale (from “strongly agree” to “strongly disagree”).

#### Medical treatment

Physicians are asked in questionnaires to provide information about their patient’s medication. This includes immunotherapy, cancer-specific therapy, chemotherapy with the aim of improving survival and response rates, and established palliative treatment and best supportive care to improve symptoms and quality of life.

#### Utilization of healthcare services

Patients are asked on the questionnaire to what extent they have utilized such services. They include rehabilitation, physiotherapy, psycho-oncological treatment, physician contacts with and without referral, care by the family physician, admission to an emergency room or hospital, duration and reason for hospitalization.

#### Disease costs

The costs of diagnostic and therapeutic measures are obtained from the participating centers (medical controlling). If they are not available in full, secondary data, for example hospital statistics, will be used.

#### Covariables

The physician questionnaire records information on the type and stage of the cancer pursuant to the UICC TNM classification. In addition to patient questionnaires, demographic information is collected on, inter alia, age, gender, educational attainment, living conditions, work situation, migration background, work incapacity, and relationship status. Finally, patients are asked about any comorbidities based on a list of conditions.

### Statistics

#### Sample size planning

The sample size planning is based on the primary endpoint, whose one-year incidence is estimated to be 25% in 2015 and 2016 according to data from the participating study centers. Based on earlier study findings, the case management program can halve the number of hospitalizations [11].

Thus, a hospitalization rate of 12.5% is expected in the intervention group. To illustrate this difference at a significance level of alpha = 5% and a statistical power of 80%, 152 patients per group are needed (chi^2^ test, normal approximation, PROC POWER SAS 9.4). Taking into account a dropout rate of 10% during the 12-month study period, a total of 170 patients per group, or 340 patients in total, are needed at baseline.

#### Statistical methods

The data are analyzed using SAS Version 9.4 (SAS Institute Inc., Cary, NY, USA). In general, the data analyses focus on the differences between the intervention group and the control group at baseline and the other data-collection times in the study. An error probability of alpha = 0.05 is defined as the significance level.

#### Descriptive data analysis

Appropriate descriptive statistical methods are applied depending on the scales used for the individual variables. Categorical variables (for example, the hospitalization rate) are expressed as absolute and relative distributions. In the case of continuous data, the following parameters are calculated: frequency, number of missing values, mean with standard deviation, minimum, maximum, median, and 25 and 75% quartiles. Suitable graphic methods are used for visualizing the data, e.g. box plots.

#### Primary endpoint

As a starting point, the hospitalization rates, defined as the percentage of patients who spend at least one night in hospital during the one-year study period in the two groups, are compared by means of the chi^2^ test. The results are verified in a second analytical step using a general linear model that takes into account other influencing factors.

#### Secondary endpoint

The differences in the HADS-S score between the intervention group and the control group are compared by means of the T test, and changes over time by means of an analysis of variance (ANOVA).

#### Other secondary and explorative endpoints

Subgroup analyses are planned, especially in respect of the cancer type (indication), the cancer state, the cancer relapse status, and the presence of metastases.

In the addition, the following subgroups are evaluated:

- Patients in employment immediately after the cancer diagnosis
- Patients in employment after the first treatment, at the start of rehabilitation
- Patients of retirement age immediately after the cancer diagnosis
- Patients of retirement age after the first treatment, at the start of rehabilitation

Other analyses and possibly tests for statistical significance are specified in the statistical analysis plan.

#### Handling of missing values

Missing values and premature withdrawals are documented. Detailed analyses are conducted of patients with incomplete data records.

The method for handling missing values depends on the standard specifications of the individual questionnaires. They differ, for example, in the number of permissible data items missing and in the method for replacing any missing data.

Two predefined intent-to-treat (ITT) populations are used for analyzing the primary endpoint:

- ITT-1: All patients who were randomized to one of the two treatment groups. If a patient dies before the end of the study, the actual hospitalization rate is used for the primary endpoint.
- ITT-2: All patients who were randomized to one of the two treatment groups. If a patient dies before the end of the study, the patient is regarded as hospitalized for the primary endpoint.
- PP: All patients for whom data are available at the time of the evaluation.

If the number of prematurely withdrawn patients differs significantly from the expected 10% limit, additional sensitivity analyses will be performed. In this case, a last-observation-carried-forward (LOCF) analysis is planned.

## Discussion

Although concepts for the care and support of cancer patients in Germany are gaining interest among decision-makers in the healthcare system, there is a lack of widespread implementation. Economic data on the implementation of such programs are essential for improving their acceptance. Programs for the care and support of cancer patients can be easily integrated into routine medical practice and can therefore also provide benefits outside clinical research.

In order to substantiate or quantify the positive effects of the support provided by patient navigators, the Saxon Cancer Society and its partners designed the prospective, randomized, multicenter, longitudinal study described below.

The study population in this evaluation comprises cancer patients with gastric carcinoma, pancreatic carcinoma, colorectal cancer, malignant melanoma or gynecological cancer who have been hospitalized at least once at one of the study centers as well as their relatives, outpatient and inpatient physicians, and cancer nurses. If randomized into the intervention group, the patients receive support from cancer patient navigation program specialists over a period of 12 months.

In terms of conduct, the data are collected on standardized paper forms. Patients, their relatives, physicians, and study nurses enter the data anonymously and without outside help. The average time required should not exceed 30–40 min per patient/relative for each data-collection point. The report forms are checked in the study center by trained data entry clerks and entered into the database. The cancer patient navigation program specialists work in accordance with the SKG’s standard operating procedures (SOPs).

Recruitment of the patients began in November 2017, and the patients were recruited over a period of six to nine months. The study is currently ongoing.

## Abbreviations

ANOVA: Analysis of variance
DRKS: Deutsches Register Klinischer Studien (German Clinical Trials Register)
HADS: Hospital Anxiety and Depression Scale
ITT: intent-to-treat
LOCF: Last-observation-carried-forward (analysis)
MOS SSS: Medical Outcomes Study Social Support Survey
PAM 13: Patient Activation Measure 13
PEF-FB: Participatory decision-making for chronic diseases
RCT: Randomised Controlled trial
SF-36: Short Form 36
SKG: Saxon Cancer Society
SOP: Standard Operating Procedure
t1, t2: Time point (1, 2)
TNM: Tumor Nodes Metastasis (Classification of Malignant Tumors system)
*UICC*: Union for International Cancer Control
US: United States
